# Optimized Design of a Self-Biased Amplifier for Seizure Detection Supplied by Piezoelectric Nanogenerator: Metaheuristic Algorithms versus ANN-Assisted Goal Attainment Method

**DOI:** 10.3390/mi13071104

**Published:** 2022-07-14

**Authors:** Swagata Devi, Koushik Guha, Olga Jakšić, Krishna Lal Baishnab, Zoran Jakšić

**Affiliations:** 1 Department of Electronics and Communication Engineering, National Institute of Technology Silchar, Silchar 788010, Assam, India; swagata_rs@ece.nits.ac.in (S.D.); koushik@ece.nits.ac.in (K.G.); klb@ece.nits.ac.in (K.L.B.); 2 Center of Microelectronic Technologies, Institute of Chemistry, Technology and Metallurgy—National Institute of the Republic of Serbia, University of Belgrade, Njegoševa 12, 11000 Belgrade, Serbia; jaksa@nanosys.ihtm.bg.ac.rs

**Keywords:** microelectronics, MEMS/NEMS, diagnostics of epileptic seizures, implantable devices, preamplifiers, piezoelectric nanogenerators, metaheuristic algorithms, artificial intelligence, neural network fitting, goal attainment method

## Abstract

This work is dedicated to parameter optimization for a self-biased amplifier to be used in preamplifiers for the diagnosis of seizures in neuro-diseases such as epilepsy. For the sake of maximum compactness, which is obligatory for all implantable devices, power is to be supplied by a piezoelectric nanogenerator (PENG). Several meta-heuristic optimization algorithms and an ANN (artificial neural network)-assisted goal attainment method were applied to the circuit, aiming to provide us with the set of optimal design parameters which ensure the minimal overall area of the preamplifier. These parameters are the slew rate, load capacitor, gain–bandwidth product, maximal input voltage, minimal input voltage, input voltage, reference voltage, and dissipation power. The results are re-evaluated and compared in the Cadence 180 nm SCL environment. It has been observed that, among the metaheuristic algorithms, the whale optimization technique reached the best values at low computational cost, decreased complexity, and the highest convergence speed. However, all metaheuristic algorithms were outperformed by the ANN-assisted goal attainment method, which produced a roughly 50% smaller overall area of the preamplifier. All the techniques described here are applicable to the design and optimization of wearable or implantable circuits.

## 1. Introduction

Millions of people around the world suffer from epileptic seizures, and more than 25% of them are resistant to any medication treatment [1,2,3,4,5,6,7,8,9]. Monitoring and prevention through neural stimulation remains a solution of choice for them. Their lives would be vastly improved if systems for real-time monitoring and electrostimulation for prevention or mitigation of seizures were to be used on a 24/7 basis [9,10,11]. From this point of view, implantable microsystems with long-life implanted power supplies appear superior over wearable systems. The implantable devices are mostly based on an amalgamation of microelectronic and micro/nano-system technologies [9,10,11].

There exist two main groups of implantable devices for epileptic seizure detection and neurostimulation, the vagus nerve stimulation (VNS) devices [9], typically implanted in the patient’s neck and much more efficient but also more invasive intracranial Deep Brain Stimulation (DBS) systems [10]. The DBS electrodes are implanted directly in the patient’s brain into one or more “hotspots” (epileptogenic zones). Both systems will have most of their circuitry built into, for example, in the patient’s chest, and their electrodes will be directly reading out/stimulating nerves responsible for the seizure onset and incorporate a preamplifier preceding a seizure detector. Typical low-power systems for epileptic seizure detection and subsequent neurostimulation have been presented by Salam et al. [11].

Implantable medical devices (IMDs) have been increasing in popularity, but the absence of long-life power sources to supply them has been observed as one of the major challenges to their widespread use in acquiring and interpreting electrical data from the human brain and nervous system [10]. The development of IMDs faces critical challenges in terms of reducing their size and expanding the device lifetime. When minimizing the IMDs, we encounter conflicting requirements, since each separate functionality expected from a detection and neurostimulation system poses a need for an additional dedicated circuit area and, hence, a dimension increase.

Among the bulkiest parts of an IMD are the implanted power sources, which may include non-rechargeable batteries that have to be periodically replaced [10], wirelessly rechargeable batteries (transcutaneous charging) [11], and self-charging nanogenerators [12].

The most advanced power supply devices are nanogenerators. Their main types include triboelectric, piezoelectric pyroelectric, and hybrid. Each nanogenerator type has its own advantages and disadvantages.

Piezoelectric nanogenerators (PENGs) are versatile micro/nano-electromechanical systems (MEMS and NEMS) which utilize the piezoelectric effect to harvest mechanical energy from the ambient and convert it into electrical energy. Their main advantages are the ease with which their dimensions can be scaled down, even to the nanometer level; the ability to merge their function with microelectronic integrated circuits; their durability; and the ease of their batch fabrication. They are extremely versatile lightweight power supply devices with an enormous number of possible applications, including such diverse fields as consumer electronics, smart textiles, optofluidic logical circuitry, home security, biomedicine, and many others [13,14,15,16]. 

PENGs are probably the best candidates for supplying implantable biomedical devices by converting biomechanical energy directly into electrical energy [17]. These devices are mechanically flexible and stretchable because they are used on soft and exceedingly deformable tissues of the human body [18,19]. As a result, it is possible to harvest biomechanical energy from natural body motions, such as muscle contractions and relaxations, cardiac and lung motions, blood circulation, and even motion caused by gravity, and supply the generated power to the electronic circuitry.

Some of the biomedical applications of PENGs are presented in References [10,11,12,17,18,19,20,21,22,23,24], for example. PENG-powered IMDs inserted directly in the brain have been suggested by some authors [11,23]. A typical implantable device for epileptic seizure detection and subsequent neurostimulation, such as that presented by Salam et al. [11], incorporates a preamplifier preceding a seizure detector. We further dedicate our attention to preamplifier circuits in the seizure detection part of the such a system.

An operational transconductance amplifier (OTA) in the preamplifier circuits needs to be properly scaled in order to meet the design target and specifications [24]. The traditional sizing process of MOSFETs [25] is time-consuming, laborious, and depends on human expertise. Thus, alternative methods that are robust and reliable, such as meta-heuristic optimization techniques, have gained popularity in solving complex circuit design problems [26,27]. Vural et al. [28] and Motlak et al. [29] have implemented particle swarm optimization (PSO) to minimize the area in a two-stage op-amp and power in a self-biased folded cascode. Moreover, there are other reported works, such as that by Kudikala et al. [30], where the harmony search algorithm (HS) and differential evolution (DE) algorithm were applied for error minimization in a folded cascode structure. Similarly, Majeed et al. [31,32] implemented a grey wolf optimization (GWO), gravitational search algorithm (GSAPSO), and hybrid whale optimization algorithm (WOA) for area minimization in two-stage op-amp and differential amplifiers.

Moreover, significant works has been performed in the field of AI-assisted optimal design of electronic circuits. A parameter optimization of a chaotic circuit by the use of Bayesian optimization and genetic algorithm has been reported by Acharya et al. [33], and regression-model-based optimization of analog mixed signal circuits has been reported by Nam et al. [34]. An overview of various AI applications for power electronics in design, control, and maintenance life-cycle phase [35] lists typical tasks (optimization, classification, regression, and data structure exploration) and methods (deterministic programming methods based on linear or quadratic programming; nondeterministic programming methods, such as metaheuristic ones) in over 500 publications [36,37]. However, in spite of all the previous works, predictions and decision-making in the optimal design of implantable electronic circuits that are capable of exchanging data with the cloud have been and still are the main focus of active research.

The objectives of the research presented in this paper are focused on the possibilities for the optimal design of a preamplifier circuit used in the monitoring part of the implantable seizure control neurostimulator. We seek the optimal set of circuit parameters that ensures the minimal overall circuit area while keeping all its functionalities intact. In order to achieve this purpose, we have advanced optimization techniques such as metaheuristic algorithms and artificial intelligence (artificial neural networks, ANNs). Furthermore, we performed quantitative and qualitative comparative analyses of the results with respect to the convergence speed and the possibility of falling into the local optima in high-dimensional space.

This paper is organized as follows: the explanation of the improvised fully differential amplifier circuit is given in Section 2, the description of the design methodology related to the use of metaheuristic optimization algorithms is given in Section 3, the description of the design methodology related to the use of the ANN-assisted goal attainment method is given in Section 4, Section 5 presents results and discussion, and Section 6 concludes the paper.

## 2. Modified Recycling Folded Cascode Amplifier (MRFC)

**Figure 1:**
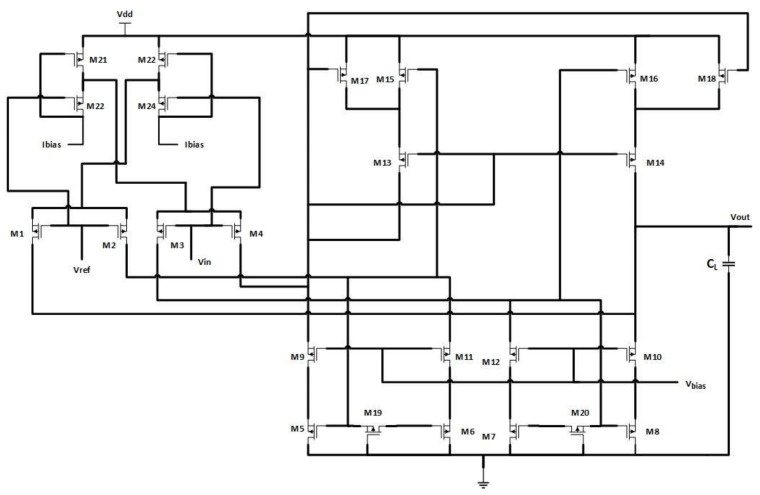
Modified recycling folded cascode amplifier.

The structure for the amplifier proposed by Devi et al. [38] is represented in Figure 1. The structure uses an adaptive biasing technique in a modified recycling folded design. The transistors M_15_ and M_16_ additionally contribute to the input drive, along with M_5_ and M_6_. To do this, a crossover link is set up between M_6_–M_15_ and M_7_–M_16_. The transistors M_17_ and M_18_ are included in the design to make it a single-ended structure, such that a current of $\frac{\left(k-1\right)}{10}I_{bias}$ flows through it. All the devices in the structure operate in weak inversion regions [39,40]. Whenever a substantial signal is employed into V_ref_, the transistors M_1_ and M_2_ are turned off, and M_4_ will function in the deep triode region of the weak inversion. The bias current conducting through M_3_ is imaged by *k* and (*k* − 1) into M_9_ and M_10_, and then into the load capacitor C_L_ by M_17_ and M_18_. The design shows improvement in slew rate as it is multiplied by a factor of (2*k* − 1) than the conventional structures [41]. There is an overall increase in transconductance, gain bandwidth product, and slew rate. Higher values of *k* lead to the degradation of the phase-margin in the design and make it unstable. To avoid this, compensation resistors realized by transistors M_c1_ and M_c2_ working in deep triode regions are inserted between the gates of the current mirror [42].

### 2.1. Drain Current Equations in Weak Inversion

The weak inversion region extends high gain and low power consumption. The specific current ($I_{S}$) in weak inversion is given by the following:

(1) $$I_{S}=K_{w}µC_{ox}\left(\frac{W}{L}\right){V_{T}}^{2},$$

and $K_{w}=2\times \eta$. Then Equation (1) is modified into the following:

(2) $$I_{S}=2\eta µC_{ox}\left(\frac{W}{L}\right){V_{T}}^{2},$$

where *η* is the aspect ratio; µ is the mobility; $C_{ox}$ is the oxide capacitance; and $V_{T}=\frac{kT}{q}$ is the thermal voltage, where *k* is the Boltzmann’s constant, *T* is the temperature in Kelvin, and *q* is the single electron charge. Then the expression of drain current for conduction in weak inversion is given by the following:

(3) $$I_{D}=K_{w}\beta {V_{T}}^{2}e^{\frac{V_{G}-V_{tho}}{\eta V_{T}}}\left[e^{\frac{-V_{S}}{V_{T}}}-e^{\frac{-V_{D}}{V_{T}}}\right],$$

where $\beta =µC_{ox}\left(\frac{W}{L}\right)$. Using the values of $K_{w}$ in (3), the equation changes to the following:

(4) $$I_{D}=2\eta \beta {V_{T}}^{2}e^{\frac{V_{G}-V_{tho}}{\eta V_{T}}}\left[e^{\frac{-V_{S}}{V_{T}}}-e^{\frac{-V_{D}}{V_{T}}}\right].$$

where η is the subthreshold slope factor and is given by $1+\frac{C_{d}}{C_{ox}}$; the threshold voltage is denoted by $V_{tho}$; and the gate, source, and drain voltages are symbolized by $V_{G},V_{S}$, and $V_{D}$ [43]. By substituting the terms of (4) with (1), the equation is modified into the following:

(5) $$I_{D}=I_{S}e^{\frac{V_{G}-V_{tho}}{\eta V_{T}}}\left[e^{\frac{-V_{S}}{V_{T}}}-e^{\frac{-V_{D}}{V_{T}}}\right].$$

Now, by considering

(6) $$I_{DO}=I_{S}e^{\frac{-V_{tho}}{\eta V_{T}}}$$

in Equation (5), we have the following:

(7) $$I_{D}=I_{DO}e^{\frac{V_{G}}{\eta V_{T}}}\left[e^{\frac{-V_{S}}{V_{T}}}-e^{\frac{-V_{D}}{V_{T}}}\right].$$

In subthreshold region, $V_{DS}>4V_{T}$, then $V_{DS}≅104\;\mathrm{mV}$. Thus, we have the following:

$$e^{\frac{-V_{DS}}{V_{T}}}=e^{-4}≅0.018<1.$$

Equation (7) can be rewritten as follows:

$$I_{D}=I_{DO}e^{\frac{V_{GS}}{\eta V_{T}}},$$

(8) $$I_{D}=I_{S}e^{\frac{-V_{tho}}{\eta V_{T}}}e^{\frac{V_{GS}}{\eta V_{T}}},$$

$$I_{D}=2\eta µC_{ox}\left(\frac{W}{L}\right){V_{T}}^{2}e^{\frac{V_{GS}-V_{tho}}{\eta V_{T}}}.$$

The $V_{DS}$ required to do so is independent on $V_{GS}$; hence, it is easy to keep the MOSFETs in saturation [44]. Therefore, the drain current equations for MOSFETs operating in weak inversion is given as follows:

(9) $$\mathrm{For}\;\mathrm{PMOS}:\;I_{D}=2\eta {µ}_{p}C_{ox}\left(\frac{W}{L}\right){V_{T}}^{2}e^{\frac{\left|V_{thp}\right|-V_{SG}}{\eta V_{T}}}.$$

(10) $$\mathrm{For}\;\mathrm{NMOS}:\;I_{D}=2\eta {µ}_{n}C_{ox}\left(\frac{W}{L}\right){V_{T}}^{2}e^{\frac{V_{GS}-V_{thn}}{\eta V_{T}}}.$$

The slew rate is given by the following:

(11) $$SR=\frac{Biascurrent}{Loadcapacitor}=2GBW\eta V_{T},$$

and

(12) $$g_{m1}=2\pi GBWC_{L}.$$

### 2.2. Adaptive Biasing Technique

The structure used an adaptive-biasing circuit in the modified recycling folded cascode to attain high gain, high bandwidth, and high slew rate conditions [45]. The currents flowing through input transistors M_1_–M_4_ are as follows:

(13) $$I_{b1}=2I_{b}e^{\frac{V_{id}}{\eta V_{T}}},$$

(14) $$I_{b2}=2I_{b}e^{\frac{-V_{id}}{\eta V_{T}}}.$$

Citing the works reported in References [39,40,45], the slew rate is given as follows:

(15) $$SR=\frac{2I_{b}\left(2k-1\right)}{C_{L}},$$

where *k* = 3, and $C_{L}$ is the load capacitance.

### 2.3. Design Procedure

Step 1: Determining the bias current, $I_{b}$, from the slew rate and load capacitance.

(16) $$I_{b}=\frac{SR*C_{L}}{2\left(2k-1\right)}.$$

Step 2: The cascode bias currents in the transistors are divided in the ratio of *k*:1, and the current through M_5_ and M_8_ is kept greater than the bias current to avoid no current through them.

Step 3: The widths of the cascode transistors can be calculated from the minimum output voltage of the circuit and the current flowing through M_5_ and M_8_. The widths of M_6_ and M_7_ can be calculated from $W_{7}=\frac{W_{8}}{k}$ and $W_{6}=\frac{W_{5}}{k}$. The current flowing through M_10_ is (2*k* − 1) times the current in M_8_, and the widths $W_{9}$ and $W_{10}$ can be estimated accordingly. The widths for transistors M_11_ and M_12_ are half those of M_9_ and M_10_ [46].

Step 4: Likewise, by taking into consideration the maximum output voltage of the circuit and the drain current equations for the PMOS transistors, the aspect ratios of transistors M_14_–M_16_ can be calculated. The transistors M_17_–M_20_ are $\frac{1}{k}$ times that of $W_{15}$ and $W_{16}$ [47,48].

Step 5: The aspect ratios for the input transistors M_1_–M_4_ can be assessed with the design parameters: gain bandwidth product and load capacitance, as illustrated in Equation (17).

(17) $$g_{m1}=2\pi GBWC_{L},$$

where $g_{m1}$ is the transconductance of the input transistor, which can be expressed as shown in Equation (18):

(18) $$g_{m1}=\frac{I_{D}}{\eta V_{T}}.$$

Step 6: The minimum and maximum input common mode range voltages, *V_in_*_(*max*)_ and *V_in_*_(*min*)_, determine the aspect ratios of the biasing and cascode transistors. The overall area occupied by the transistors can be computed from Expression (19), where *n* is the maximum number of transistors involved, and $L_{n}$ is the length of the *n*th transistor [41].

(19) $$Area=\sum_{n}W_{n}L_{n}.$$

## 3. Meta-Heuristic Optimization Algorithms

In analog circuits, several kinds of variables, objectives, constraint functions, and variables are exercised. Their effectiveness is substantially reliant on the number of variables, defined parameters of algorithms, the size, and convexity of solution. The heuristic algorithms, such as local search (LS), simulated annealing (SA), and many more, provide deterministic and inexact approximate solutions [49]. However, they mostly fail to deliver generalized solutions involving objectives and constraints. Hence, these optimization methods may prove themselves inadequate. Thus nature-inspired heuristic optimization algorithms, better known as metaheuristic algorithms, are employed instead [50]. These techniques are versatile, efficient, and easy to use. They are Swarm Intelligence algorithms, and they emphasize the behavior of an animal or insect to cultivate a few metaheuristics that are capable of imitating their problem-solving skills [51].

**Figure 2:**
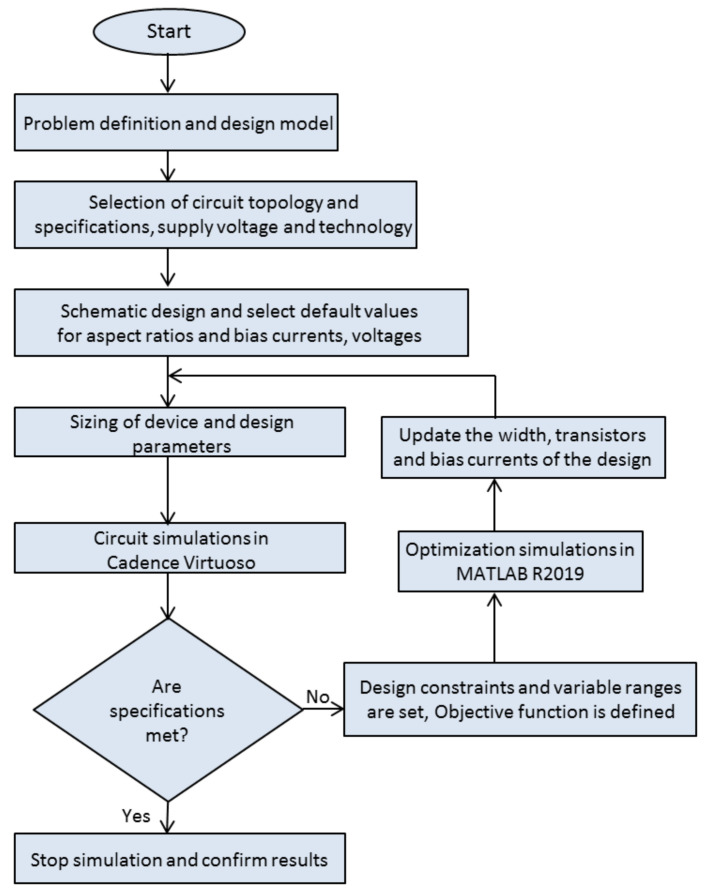
Process flow for the metaheuristics considering circuit optimization.

Among others, the static and dynamic swarming activities of the dragonflies in natural surroundings were mimicked. The analytical design for exploration and exploitation of the Dragonfly Optimization Algorithm (DOA) was modeled by studying the social communication of these species which includes piloting, food searching, and avoiding foes [52]. The exploration phase is the imaging of static swarms, where they form sub-swarms and hover over diverse areas; meanwhile, in the exploitation phase, the movement of bigger swarms along one direction is mimicked [53]. In the Grasshopper Optimization Algorithm (GOA), a swarm of grasshoppers mimics the behavior of the nymph and the adult, where the former jumps and moves similar to a rolling cylinder, and later they form a swarm in the atmosphere and wander over huge distances. Food-source seeking is another important characteristic of the swarming of grasshoppers [54]. In the grey wolf optimization (GWO), the leadership hierarchy and hunting mechanism of the grey wolves are mimicked [55]. In the hybrid GWO, the GWO algorithm was hybridized with particle swarm optimization to increase the performance of GWO. Particle swarm optimization (PSO) is a swarm-based method where the optimum solution of the problem is established by observing the motion of particles in search space. The advantages of the hybrid GWO–PSO algorithm are its simplicity and faster convergence in finding solutions to global optimization problems [56]. The Mayfly Optimization Algorithm (MOA) is designed by mimicking the mating process in mayflies. It unites the prime benefits of existing algorithms and is inspired by the behavior of adult mayflies, including the practices of the swarm [57]. Crossover, mutation, and gathering establish the exploitation phase, while nuptial dance and random walk enhance exploration. The motivation of the Marine Predator Optimization (MPO) algorithm is the foraging strategy exhibiting the Lévy and Brownian activities of ocean predators. It includes the optimal encounter rate strategy in biotic practices between predator and prey [58]. These rules inherently encompass the optimal foraging style and encounter rate policy-relating predator and prey in marine ecosystems. The whale optimization algorithm (WOA) is inspired by the hunting behavior of humpback whales. It imitates the three mechanisms, the search for prey, encircling prey, and bubble-net foraging behavior of humpback whales [59]. A general process flowchart for the metaheuristic approach is shown in Figure 2.

## 4. ANN-Assisted Goal Attainment Method

**Figure 3:**
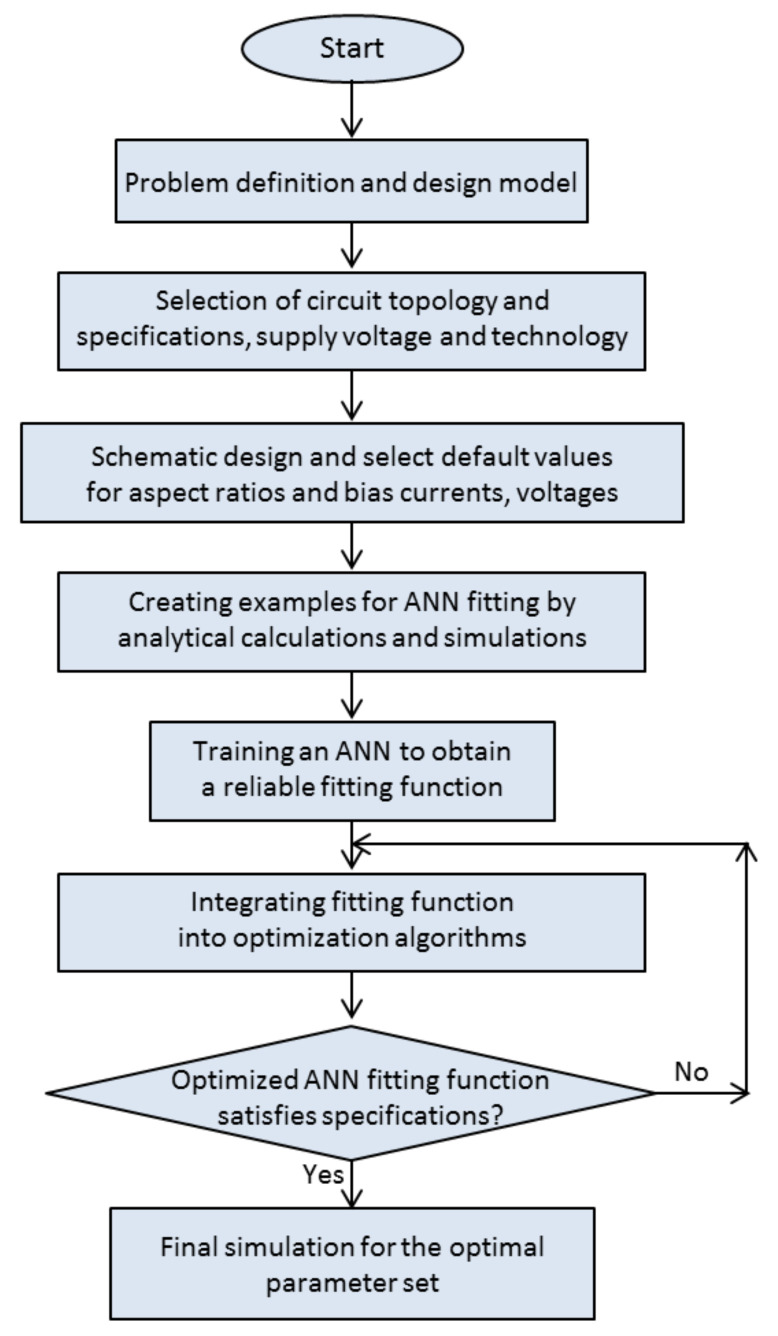
Flow diagram summarizing the protocol for determining the optimal set of circuit parameters that ensures minimal area.

The protocol that was used for the estimation of optimal circuit parameters by the use of neural network fitting and goal attainment method is shown in the flowchart in Figure 3.

The process of supervised training of an artificial neural network starts with the creation of the dataset of examples for feeding the ANN. The dataset that was used for training the ANN was created by conditioning the data from 150 numerical simulations performed in the Cadence 180 nm SCL environment. For every example intended to be fed to the ANN, a fixed point in the parameter space is chosen. The parameter space is formed by the following circuit parameters: slew rate, load capacitor, gain–bandwidth product, maximal input voltage, minimal input voltage, input voltage, reference voltage, and dissipation power. These values are first used for the area calculations as per Equations (1)–(19) and then, additionally, for simulations in Cadence environment, which generates the values for gain, phase, noise, power, bandwidth, and area and ensures that the design is in accord with the technology. The area is then stored as a target output value for the chosen example. Then the discriminative neural network, which is capable of predicting the overall circuit area based on the eight numbered inputs (circuit design variables), is created in order to provide the fitting function that replaces numerical simulations and that can be integrated in the optimization algorithm, which is, here, contrary to metaheuristic ones, a deterministic one, a goal attainment method, based on the sequential quadratic programming.

The approach shown by the flowchart in Figure 2, where the ANN serves as an approximate replacement for analytical equations or circuit simulations used to calculate the cost function formula, is also suitable for combinations of ANN fitting functions and metaheuristic algorithms. The main advantage of using an ANN in that case is in its speed, and it is, thus, most practical for higher complexity circuits and circuit models. Fitting functions are simple and suitable for calculations scaled for a greater or more dense parameter space. It is possible that the parameter set delivered by using metaheuristic algorithms, after the final simulation, can lead to a circuit with a greater area than that obtained by parameter sets delivered by the goal attainment method (or any other method based on the deterministic search).

### 4.1. ANN Fitting of the Overall Circuit Area

**Figure 4:**
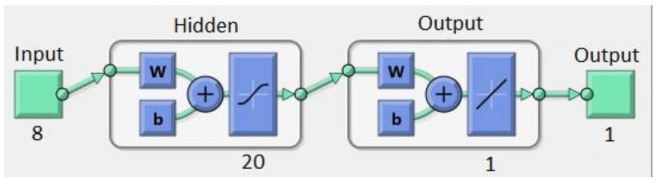
Structure of the ANN used for fitting the circuit area.

The artificial neural network that we used for fitting the relation between the overall circuit area and the eight related parameters was a shallow forward-feed backward propagation network whose structure is shown in Figure 4. Since the dataset has overall 150 examples, the proposed ANN is shallow, having a single hidden layer. In the case of a greater number of available datasets, a deep structure, one with several hidden layers, may be used [60]. The way to seek for the best ANN configuration, for a predefined list of device variables, inputs, and outputs, is by adding more hidden layers (and applying the deep learning techniques [61]), by changing the number of neurons in hidden and output layers, or by changing the activation function. Among various possible activation functions, the Sigmoid Symmetric Transfer Function was used in this work:

(20) $$y=\frac{2}{1+e^{-2x}}-1.$$

For solving the problem described in this paper, two ANN structures were used, one with 10 nodes in a hidden layer, and the other with 20 nodes in a hidden layer. Both ANNs had the same eight inputs corresponding to slew rate, load capacitor, gain bandwidth product, maximal input voltage, minimal input voltage, input voltage, reference voltage, and dissipation power. The same iterative training process for both of them was performed in the same way.

Before feeding the data to the ANN, the initial dataset is divided stochastically into three groups: the training set of examples that “teaches” the ANN and “shows” proper answers to ANN in every iteration; the validation set of examples that serves for the comparisons between the values predicted by the ANN and the values that corresponded to true values given in examples in the validation set, also in every iteration; and the testing dataset of examples. The testing dataset is used only after the iterative process of ANN adaptation ends.

In the process of creating the ANN structure, various ratios for the data division are explored. There is no rule for an ideal ratio. In general, the importance of the division is greater for small datasets. Larger training sets will be better for smaller example datasets. It is also important to observe if there are any favorable data intervals and to ensure that there are sufficient examples in relevant training and validation subsets related to them.

In every iteration, the sigmoid function of the biased and weighted sum of inputs is computed in all nodes in the hidden layer. The linear function of the biased and weighted sum of the outputs of the hidden layer is computed in the output layer. Weights and biases iteratively change until the desired similarity between the output generated by the ANN and the original output, as per examples (provided by Cadence simulations), is met.

The quality of the ANN fitting is evaluated through *MSE* (Mean Squared Error) and regression values. The *MSE* is calculated as follows:

(21) $$MSE=\frac{1}{N}\sum_{i=1}^{N}{\left(e_{i}\right)}^{2}=\frac{1}{N}\sum_{i=1}^{N}{\left(t_{i}-a_{i}\right)}^{2}.$$

where *N* is the number of examples (input–output pairs) used for training the network; *t* is the target value, and here it is the circuit area as per Cadence simulations; *a* is the value predicted by the ANN; and *e* is the error, i.e., the difference between the target value and the value predicted by the ANN.

The regression, *R*, is related to the coefficient of the determination, *R*^2^, an indicator of the correlation between the target values and the values predicted by the ANN, and it is calculated as follows:

(22) $$R^{2}=1-\frac{\sum_{i=1}^{N}{\left(t_{i}-a_{i}\right)}^{2}}{\sum_{i=1}^{N}{\left(t_{i}-\underline{t_{i}}\right)}^{2}},$$

with the same notation as for the *MSE* calculation, and $\underline{t_{i}}$ is the arithmetic mean of the target values. The closer *R* is to 1, the better the prediction of the ANN is.

In every iteration, the ANN adapts itself by altering biases and weights so that their combination ensures better prediction of target values, and the iterative process terminates successfully in the case of six consecutive validation checks. Behind each of the different learning algorithms lies different reasoning for finding the optimal values of the ANN parameters, its biases, and its weights.

The Levenberg–Marquardt learning algorithm has been developed to solve the least squares curve-fitting problems, as first reported by Levenberg [62] and then rediscovered by Marquardt [63]. The reasoning behind it is as follows: performance function has the form of a sum of squares—Equation (21). Then the Hessian matrix, **H**; the gradient, g; and the update for the solution, ***X***, can be approximated as follows:

(23) $$H=J^{T}\cdot J;\;g=J^{T}\cdot e$$

(24) $$X_{k+1}=X_{k}-{\left[H+\mu I\right]}^{-1}\cdot J^{T}\cdot e$$

where **J** is Jacobian, **I** is the unit matrix, and µ is scalar. When µ is zero, (24) retards to Newton’s method, which uses an approximate Hessian matrix. For a large µ, this becomes gradient descent with a small step size.

The validation data are used to stop training when any of the following conditions are met: the maximum number of epochs (repetitions) is reached; the performance is minimized to the predefined goal; the performance gradient drops below the predefined value; µ exceeds the predefined value; and the number of failed validation checks exceeded the predefined value.

When ANN parameters are far from their optimal value, the sum of the squared errors is reduced by updating the parameters in the steepest-descent direction, and when ANN parameters are close to their optimal value, the sum of the squared errors is reduced by assuming that the least squares function is locally quadratic in the parameters. After finding the ANN fitting function that reliably replaces the numerical simulations and calculates with sufficient accuracy the circuit area for input vectors made of design variables (circuit parameters), the next step is to locate the minimum of that function in parameter space.

### 4.2. Goal Attainment Method

The goal attainment method is a method developed for solving the nonlinear programming problems, such as the multi-objective optimization and sequential quadratic programming (SQP). For a set of objectives, *F_i_*(*x*), and a set of their respective design goals, $F_{i}^{g}$, the unscaled goal attainment problem is to minimize the maximum of $F_{i}\left(x\right)-F_{i}^{g}$. In a generalized form, after introducing the set of weights, $w_{i}$, the goal attainment problem aims for to find *x* while trying to minimize the maximum of the following:

(25) $$\frac{F_{i}\left(x\right)-F_{i}^{g}}{w_{i}},$$

simultaneously satisfying various constraints that can be defined by equations such as the following:

(26) $$c\left(x\right)\leq 0;\;ceq\left(x\right)=0;\;A\cdot x\leq b;\;Aeq\cdot x=beq\;\mathrm{or}\;l\leq x\leq u$$

The figure of merit of this optimization, as provided in the MathWorks MATLAB environment, is the attain factor, *a*, which is a value related to the percentage of the objectives that may be overachieved (in which case, the attain factor is negative) or underachieved (in which case, the attain factor is positive). The closer this figure of merit is to zero, the better the optimization results will be.

Applied to one objective, this method finds its minimum with respect to constraints.

## 5. Results and Discussion

### 5.1. Results of Metaheuristic Algorithms

In this work, the aforementioned algorithms were executed to minimize the die area for the optimized circuit design. The algorithm parameters that were used for minimizing the mono-objective function are described below:

1. In the Dragonfly Optimization Algorithm (DOA), the explorative and exploitative activities can be accomplished through the parameters: separation (s), alignment weight (a), cohesion weight (c), food factor (f), and enemy factor (e). These are dependent on the maximum number of iterations, which is considered to be 100 for a variable dimension of 8 and a search agent number of 80.
2. In Grasshopper Optimization Algorithm (GOA), the exploration and exploitation phase are controlled by the coefficient “c” and are dependent on the number of iterations, 100 and with search agents of 50; c_max_ and c_min_ are the maximum and minimum values that are selected as 1 and 0.00004.
3. In both the grey wolf optimization (GWO) and hybrid particle swarm optimization–grey wolf optimization (PSO–GWO), the number of search agents is 30 for a dimension of 8, while A and C are the coefficient vectors. However, in PSO–GWO the particle swarm algorithm parameters are also employed. Both the social learning and cognitive learning coefficients are kept as 0.5.
4. In the Mayfly Optimization Algorithm (MOA), the male, female, and offspring population size for mayfly swarm agents is 20 each, and the inertia weight and weight damping ratio are taken as 0.8 and 1. The personal learning, global learning, and distance sight coefficients are selected as 1, 1.5, and 2. Moreover, nuptial dance, random flight, damping ratio, and mutation rates are 5, 1, 0.8, 0.99, and 0.01.
5. In the Marine Predators Optimization Algorithm (MPOA), the value of the drifting Fish Aggregating Device (FAD) is kept as 0.2. P is a constant number and is equal to 0.5; the size of the search agents is 25, and the dimension is 8.
6. In the whale optimization algorithm (WOA), the parameters a, l, and p are random numbers in the ranges [0, 2], [–1, 1], and [0, 1]. A and C are coefficient factors. The number of search agents is considered to be 200 for a dimension of 8 and iteration value of 100.

**Figure 5:**
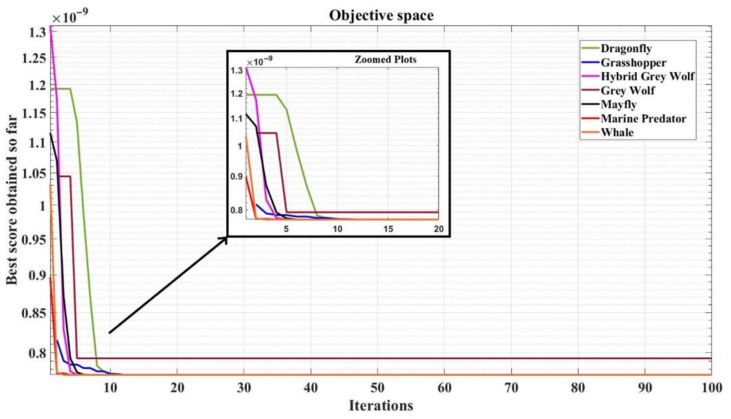
Convergence plots for the implemented metaheuristic algorithms.

The convergence plots for the algorithms, namely DOA, GOA, PSOGWO, GWO, MOA, MPOA, and WOA, are shown in Figure 5. The MPOA algorithm converges at a fastest rate, close to about an iteration number of 3. A majority of the algorithms used have been developed recently to assure a better convergence speed.

The MATLAB simulations were carried out by taking the parameters in Table 1 as variables. Table 1 also defines the ranges for each of them. Table 2 shows the values for the design constants for the circuit design problem in weak inversion region, Table 3 lists the values of the design variables for each optimization algorithm, and Table 4 shows the error percentage of the design parameters for the algorithms. It can be observed from the convergence graph and the comparison table that the Marine Predator Optimization Algorithm gives the lowest value for area and converges fastest. The grey wolf optimization (GWO) shows the worst convergence and settles at a higher value for the die area. The error percentage is greater compared to the rest of the algorithms. Moreover, Table 5 draws comparisons with some recent works on minimizing the area of the designed circuits.

**Figure 6:**
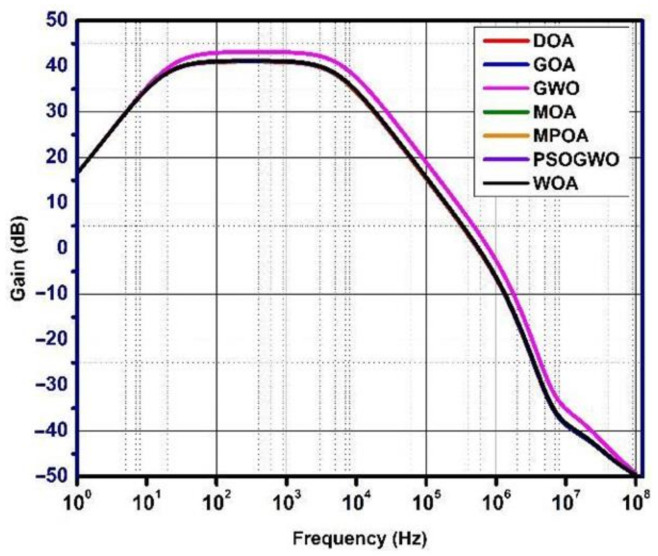
Gain vs. frequency plot for metaheuristic algorithms.

**Figure 7:**
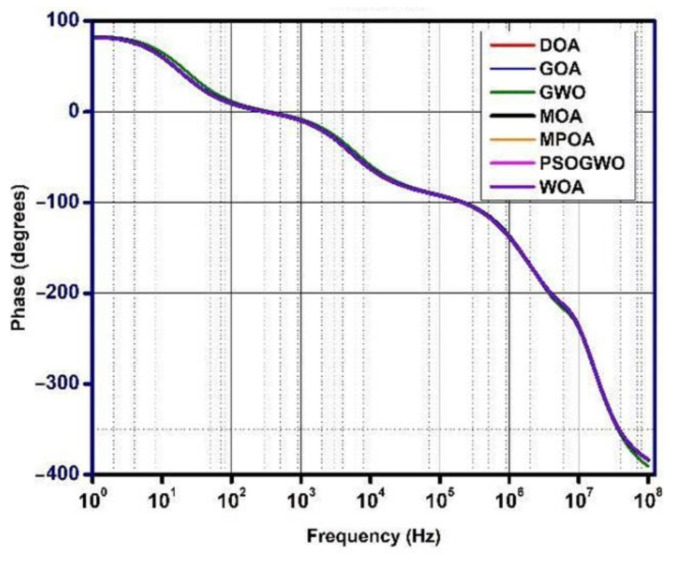
Phase vs. frequency plot for metaheuristic algorithms.

**Figure 8:**
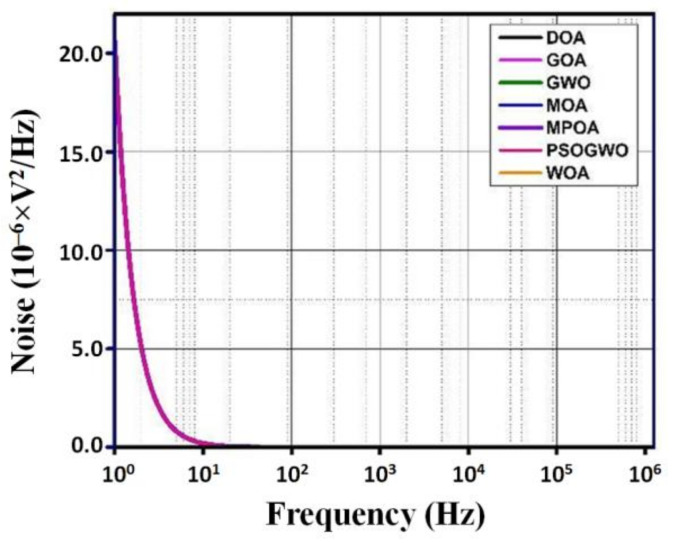
Noise vs. frequency plot for metaheuristic algorithms.

The gain, phase, and noise vs. frequency plots are shown in Figure 6, Figure 7 and Figure 8. The gain curve for the GWO algorithm is estimated to be around 43.46 dB, which is higher than what was obtained by the rest of the algorithms; however, it costs a higher die area. The phase and noise plots are mostly similar for all the cases illustrated in the diagrams.

### 5.2. Results of ANN-Assisted Goal Attainment Method

The results that are described in this section were obtained by scripts and functions written in Octave, release 6.2.0, and MathWorks MATLAB, release R2015a. In addition, the MATLAB neural network fitting application was used. The parameters used for the training were 1000 for the maximum number of epochs, 0 for the performance goal, 10^−7^ for the min performance gradient, and μ starts from 0.001, with a decrease factor of 0.1, increase factor of 10, and a maximum of 10^10^. The maximum number of failed validation checks is six.

The goal of the ANN-assisted goal attainment method was the same as the goal of metaheuristic algorithms to find optimal numerical values in a set of design variables ranged as per Table 1. However, in numerical simulations performed for the generation of examples aimed at the creation of ANN fitting functions, some ranges were broader in order to find a better fit for such a nonlinear change of the overall circuit area in a high-dimensional parameter space and ensure good generalization capabilities of the network fitting function. The slew rate was varied from 0.5 to 10 µV/s, the load capacitor was varied from 0.5 to 20 pF, the maximal input voltage was varied from 0.1 to 0.6, the minimal input voltage was varied from −0.6 to −0.1, and the input voltage was varied from 400 to 600.

Based on the extensive dataset gathered from numerical simulations, two ANN fitting functions were created. One was created by the shallow forward-feed backward propagation network with a hidden layer of 10 neurons, and the other was created by the shallow forward-feed backward propagation network with 20 neurons in a hidden layer. Both ANN fitting functions were obtained by the Levenberg–Marquardt learning algorithm for nonlinear least-squares curve-fitting problems with random data division, such as 70:15:15, meaning that, out of 150 examples in a dataset, 70% (104) were used for training, 15% (23) for validation, and 15% (23) for testing. Moreover, both ANN fitting functions are available for download from the open online repository Mendeley Data [69], along with the script for generating other ANN fitting functions (by varying the proportions for data division, the number of neurons, and the learning algorithm). The input vector is a 1 × 8 vector of numbers that correspond to slew rate, load capacitor, gain bandwidth product, maximal input voltage, minimal input voltage, dissipation power, input voltage, and reference voltage, in that respective order. Hence, the set of 150 examples aimed for feeding the ANN in the training process had a 150 × 8 matrix as an input and a column of 150 numerical values as an output.

The process of training the ANN fitting function with 20 neurons took one second to meet the training criteria by reaching six successful validation checks and the gradient drop from 2.26 × 10^6^ to 396.1957

**Figure 9:**
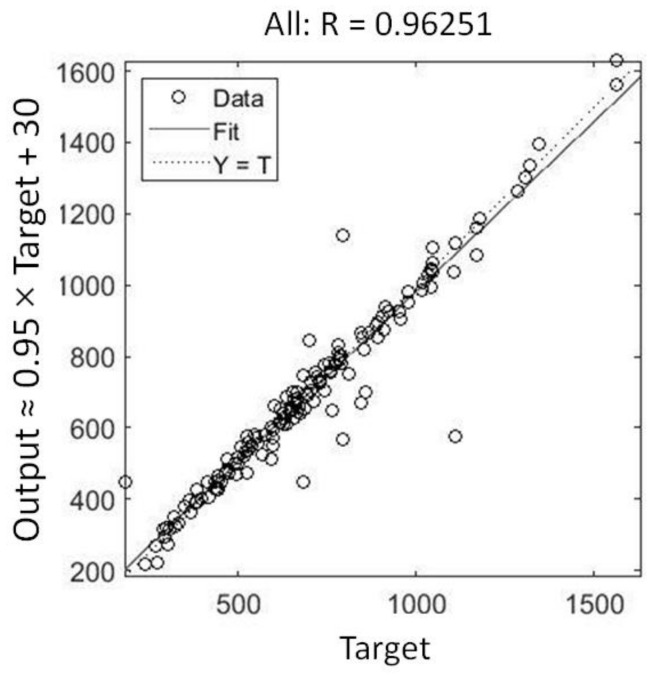
Regression of data of the whole dataset for the ANN fitting function with 20 neurons.

The regression of predicted values with respect to targets in the undivided dataset is shown in Figure 9.

The results obtained after applying the goal attainment method are presented in Table 6 and Table 7.

Different parameter sets were obtained by using different settings. For all of them, the starting point was Set 1. The constraints were the same, as defined in Table 1. For Sets 2 and 3, the goal was to minimize the ANN fitting function trained on a structure with 10 nodes in a hidden layer, and for all other sets, the goal was to minimize the ANN fitting function trained on a structure with 10 nodes in a hidden layer. For some of them, the goal was set to be close to the realistic one, such as, for instance, 170 (set 9) or 189 (set 8). Setting the goal to an unrealistically low circuit area resulted in the obtainment of Sets 2–7. The fact that the search for the minimum of the objective function was successful even in the case of an unrealistic goal revealed and confirmed the complex interplay of the relations between the circuit parameters. In spite of the good quality of the ANN fitting functions (in terms of the coefficient of the determination), due to strong nonlinearities in such a high-dimensional parameter space, wrong predictions of the circuit area were made. Thus, ANN fitting is used just as a function that can be incorporated into the procedure of the goal attainment method, and not as a tool that can replace the simulations or fabrications themselves. After the exploratory analysis of the parameter sets obtained by the goal attainment method, one final round of the simulations was performed for the circuit area comparison (last row in Table 6).

**Figure 10:**
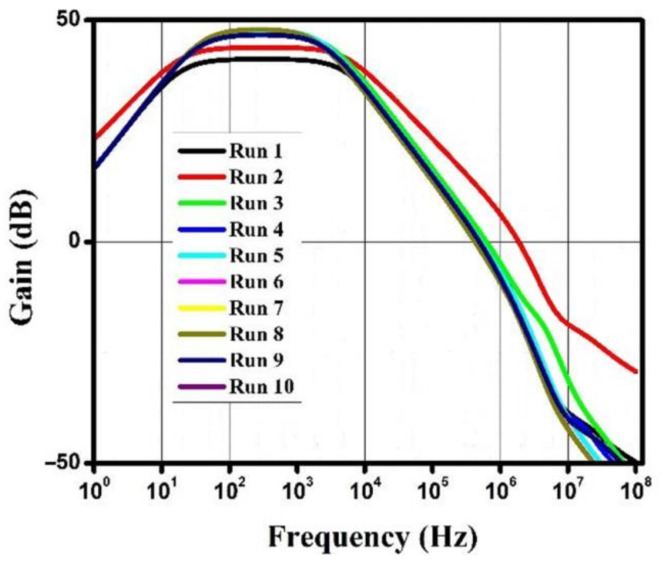
Gain vs. frequency plot for circuits designed after results from ANN-assisted goal attainment method.

**Figure 11:**
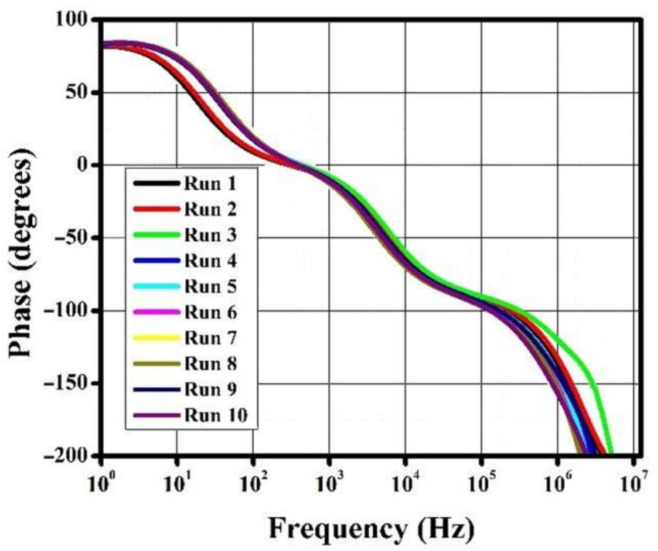
Phase vs. frequency plot after results from ANN-assisted goal attainment method.

**Figure 12:**
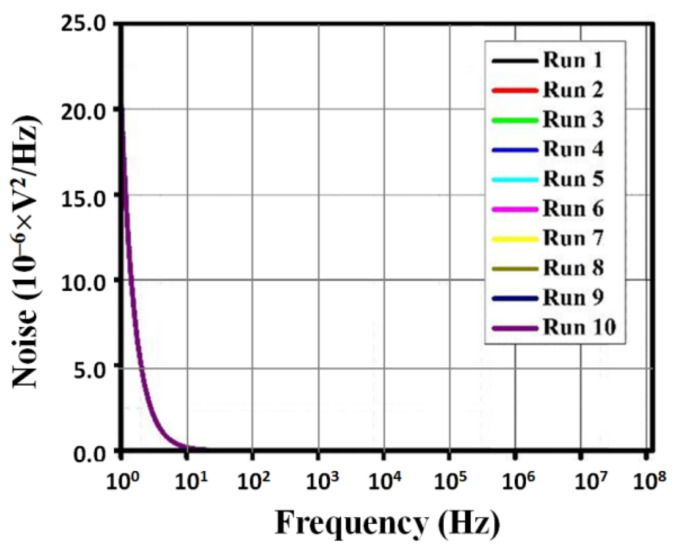
Noise vs. frequency plot after results from ANN-assisted goal attainment method.

The results for the different parameter values are represented under each set in the table. It could be observed that, for the ANN-assisted goal attainment method, Set 4 showed the best values for gain and phase, around 47.7046 dB and 46.321 degrees, respectively. The area for the design, proved by Cadence simulations, is 369.98 µm^2^. Set 8 and Set 9 deduce lower value for area in comparison to Set 4; they are 357.55 and 287.24 µm^2^, respectively. Set 8, however, has a power of 6.763 µW, which exceeds the circuit design limit of 5 µW. Set 9 consumes a power of 3.7032 µW, but the power consumed by Set 4 is lowest (815.62 nW). Therefore, considering all the parameters, Set 4 shows the best value with minimized area. Figure 10, Figure 11 and Figure 12 show a comparative representation of gain, phase, and noise plot for various sets by the ANN-assisted goal attainment method.

### 5.3. Comparative Analysis of Optimization Results

In this work, it could be observed from the results that the ANN-assisted goal attainment method has outperformed the metaheuristic algorithms. The MPOA algorithm has shown the best value for area, which comes around 773.6955 µm^2^, and provides a gain and phase of 41.255 dB and 61.96 degrees. In contrast to the metaheuristic optimization methods, the ANN-assisted goal attainment method provided better results. Based on the comparative analysis among the presented methods, it can be observed that the results in Set 4 show an improvement in terms of gain, power, and area. The goal to minimize the objective function (area) is achieved with better outcomes by the ANN-assisted goal attainment. A comparison of the results is shown in Table 8.

In the course of our research, besides the above-described algorithms, we have actually developed optimization programs for a number of other algorithms. However, we have not obtained adequate results from those algorithms. Only the best results were compared in the paper. It is noticeable that the results we chose to present do not include a genetic algorithm (GA), although a well-designed GA may be able to reach excellent optimization results (see Reference [70], for example). However, considering the No-Free-Lunch Theorem for search (NFL) [71], it is also evident that if a search algorithm performs particularly well on one set of objective functions, it can simultaneously perform poorly on objective functions for other defined problems. For the specified objective in this work, we were unable to attain the desired specification by GA, and the obtained areas were around 1271 µm^2^, which was unacceptably high.

During our research, we also incorporated several obtained ANN fitting functions into the GA; however, the global minimum was missed, and the obtained parameter set led to a circuit with an unacceptably large area. Thus, instead of insisting on finding new settings for the genetic algorithm, we decided to present this result with an aim to assist researchers with a tool that is fast and reliable. We did not challenge the superiority of metaheuristic algorithms over the deterministic ones in finding the global minima.

### 5.4. PENG Supply

Regarding autonomous power supply of the implantable neurostimulation circuitry using piezoelectric nanogenerators (PENGs), we considered a number of the existing solutions. Among those, we chose as a specific example the use of PENG harvesters in deep brain stimulation as described in Reference [72]. The dimensions of the PENG were 1.7 cm × 1.7 cm, and the obtained current was 283 µA at a voltage of 11 V. The PENG application in vagus nerve stimulation has been presented by Zhang et al. in Reference [73], with a power of 23.94 µW/cm^2^. Generally speaking, however, while PENG appears to ensure sufficient power for some specific functionalities of implantable seizure control devices, it leaves much to be desired in regard to enabling more complex multifunctionalities. A solution to consider is a combination of a rechargeable battery and a PENG.

**Figure 13:**
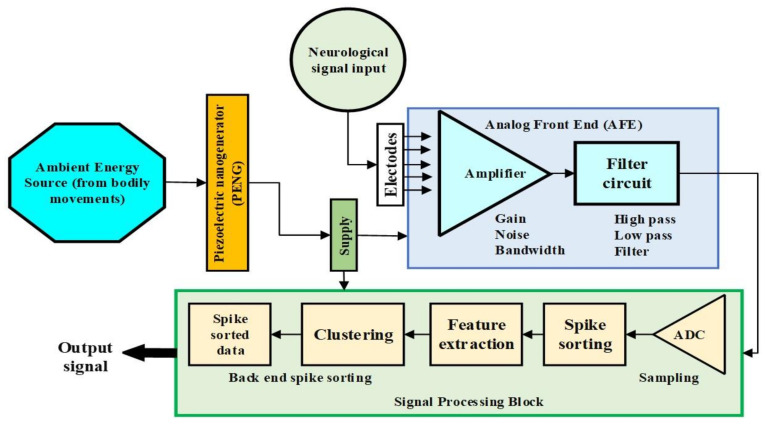
Schematic diagram of PENG supply for the analog front end (AFE) and signal processing block in a neurostimulator.

To better illustrate our approach, here we present a schematic diagram of our proposed application (Figure 13), outlining our method for supplying the neurostimulation circuits. It can be seen how the PENG is connected into the overall scheme, supplying the complete analog front end and signal processing block circuitry.

## 6. Conclusions

The work implements PSO, GWO, Hybrid PSOGWO, WOA, and DA metaheuristic algorithms to find the optimum values of circuit parameters. The aspect ratios and the biasing currents for the circuit are determined. The whale optimization algorithm (WOA) shows optimized values at an early iteration step and hence converges fastest among the implemented metaheuristic algorithms. Thus, it proved itself to be a favorable solution in optimizing the complex analog circuit sizing by metaheuristics. Furthermore, it computes at high-speed with high reliability and consumes less time than the traditional design technique. Moreover, optimization methods confirm convergence to global optimum, while the traditional methods sometimes fail to the same. Area optimization is essential for the implantable devices. Metaheuristic algorithms are an efficient approach to design the circuit with optimum values.

In comparison to metaheuristic algorithms, the protocol based on the machine-learning-assisted circuit optimization by the goal attainment method supports previous results on successful automated machine-learning-assisted electronic circuit design. The obtained circuit parameters ensure a smaller overall circuit area than the area calculated by using metaheuristic algorithms by more than 50%. In this approach, ANN serves as an approximate replacement for analytical equations or circuit simulations used to calculate the cost function formula. Further improvements might be possible by exploring different learning techniques for the creation of the neural network fitting function (apart from Levenberg–Marquardt technique used here). The minimization of the ANN fitting function, performed here by the deterministic Goal Attainment Method, can also be performed by metaheuristic algorithms. The main advantage of using an ANN to approximate the background circuit model is its speed; thus, it is most practical for higher complexity circuits. Independent of the optimization described here, ANN fitting functions can also be used in hyper-physics system models and in multiscale modeling. 

All techniques described in this work are applicable to the design and optimization of preamplifiers in implantable circuits for seizure detection. The solutions presented here aim to assist researchers with a fast and reliable tool rather than to challenge the superiority of metaheuristic algorithms over the deterministic ones in finding the global minima.

Supplying power to implanted seizure control devices at the current stage of development is achievable by a combination of a rechargeable battery and a flexible piezoelectric generator. However, PENG devices are being developed at a rapid pace, thus pointing to a very realistic possibility of fully autonomous implanted systems with complex multifunctionalities, simultaneously enabling seizure detection, neural stimulation to prevent seizure, and contact between the seizure control device and a cloud via a cellphone.

## Figures and Tables

**Table 1:** Ranges for the design variables.

Parameters	Ranges
Slew rate (V/µs)	1 to 10
Load capacitance (pF)	5 to 10
Gain bandwidth product (MHz)	1 to 10
Maximum input voltage (V)	0.2 to 0.4
Minimum input Voltage (V)	−0.4 to −0.2
Power (µW)	1 to 5
Input voltage (µV)	500 to 600
Reference voltage (mV)	1 to 2

**Table 2:** Specifications for the circuit design.

Parameters	Value
Subthreshold slope, η	1.3
Supply voltage	0.6 V
Threshold voltage, V_t_	−0.42 V, 0.42 V
Thermal voltage, V_T_	26 mV
For NMOS λ_n_	0.04 V^−1^
For PMOS λ_p_	0.05 V^−1^
Maximum output voltage	0.3 V
Minimum output voltage	−0.3 V
For NMOS, K_n_ (µ_n_ C_ox_)	355 × 10^−6^ mA/V^2^
For PMOS K_p_ (µ_p_ C_ox_)	75 V × 10^−6^ mA/V^2^

**Table 3:** Comparison of design variables for the implemented algorithms.

Parameters	DOA	GOA	PSO GWO	GWO	MOA	MPOA	WOA
Slew rate (V/µs)	1	1	1	1	1	1	1
Load capacitance (pF)	10	10	10	10	10	10	10
Gain bandwidth (MHz)	2	2	2	2	2	2	2
Maximum input voltage (V)	0.24023	0.28355	0.4	0.4	−0.20774	0.25181	0.2
Minimum input voltage (V)	−0.39493	−0.22107	−0.2	−0.4	−0.3163	−0.3163	−0.30419
Power (µW)	1	1	1	3	1	1	1
Input voltage (V)	538.1976	500	500	600	500	566.127	500
Reference voltage (V)	1011.664	1000	1000	1100	1000	1065	1000
Area (µm^2^)	773.71	773.70	773.71	793.22	773.695	773.695	773.71

**Table 4:** Validation with cadence simulations.

**Parameters**	**GWO**	**% Error**	**MPOA**	**% Error**	**DOA**	**% Error**	**GOA**	**% Error**	**Cadence Simulation**
Gain	43.16	4.13	41.255	0.47	41.022	1.03	41.135	0.76	41.45
Phase	53.64	13.82	61.96	0.45	62.597	0.57	60.13	3.39	62.24
Noise	20.63	0.34	20.558	0.01	20.616	0.27	20.617	0.28	20.56
Power	2.83	0.60	2.884	1.37	2.834	0.39	2.865	0.70	2.845
Bandwidth	6.13	15.66	5.308	0.15	5.148	2.87	5.274	0.49	5.3
Area	793.18	3.32	773.6955	5.7	773.6991	5.69	773.6956	5.69	820.38
**Parameters**	**WOA**	**% Error**	**PSOGWO**		**% Error**		**MOA**	**% Error**	**Cadence Simulation**
Gain	41.258	0.46	41.24		0.51		41.231	0.53	41.45
Phase	61.4	1.35	61.23		1.623		60.7	2.47	62.24
Noise	20.62	0.29	20.562		0.01		20.6	0.19	20.56
Power	2.87	0.88	2.839		0.21		2.834	0.39	2.845
Bandwidth	5.3088	0.17	5.3		0		5.3088	0.17	5.3
Area	773.697	5.69	773.6964		5.69		773.6988	5.69	820.38

**Table 5:** Comparison with other relevant works.

References	Gain (dB)	Phase (degrees)	Power (µW)	Noise (nV^2^/Hz)	Bandwidth (kHz)	Area (µm^2^)	Technology
Wattanapanitch et al. (2007) [64]	40.85	-	7.56	41.95	5.32	3687.84	180 nm
Chaturvedi et al. (2011) [65]	37	-	1.5	65.73	7	1044	130 nm
Ruiz-Amaya et al. (2015) [66]	46	-	1.92	44.17	7.4	1077.46	130 nm
Kim et al. (2018) [67]	39.2	49	2.4	67	28	2689.3	180 nm
Gupta et al. (2021) [68]	45.88	-	2.39	16.13	340	770.4	180 nm
This work	41.26	61.96	2.884	20.558	5.308	773.6955	180 nm

**Table 6:** Analysis from ANN-assisted goal attainment method.

	**Set 1**	**Set 2**	**Set 3**	**Set 4**	**Set 5**
Slew Rate (µV/s)	1	0.9824	2.9	1.2	4.1
Load capacitor (pF)	10	10.0181	5	5	5
GBW (MHz)	2	1.8586	1	1	1.4
Vin_m ax(V)	0.2077	1.0787	0.4	0.4	0.4
Vin_m in (V)	−0.3163	−0.9466	−0.2	−0.2	−0.2
Pdiss (µW)	1	1.1667	1.4	1	1
Input Voltage (µV)	500	500.0023	500.2	500	500
Reference Voltage (µV)	1000	999.9999	1000	1000	1000
Area (µm^2^)	781.49	746.15	425.73	369.98	513.38
	**Set 6**	**Set 7**	**Set 8**	**Set 9**	**Set 10**
Slew Rate (µV/s)	4.3	4.8	0.8	3.5	8.266
Load capacitor (pF)	5	5	5	5.4	5
GBW (MHz)	1.2	1.2	1	1.1	1
Vin_m ax(V)	0.4	0.4	0.4	0.4	0.4
Vin_m in (V)	−0.2	−0.2	−0.2	−0.2	−0.2
Pdiss (µW)	1	1	1	1	1
Input Voltage (µV)	500	500	500	500	500
Reference Voltage (µV)	1000	1000	1000	1000	1000
Area (µm^2^)	494.26	510.89	357.55	287.24	640.042

**Table 7:** Cadence simulation results for designs resulting from the use of the ANN-assisted goal attainment method.

	**Set 1**	**Set 2**	**Set 3**	**Set 4**	**Set 5**
Gain (dB)	41.187	42.273	46.7917	47.7046	47.7658
Phase (degrees)	63.119	63.02	59.33	46.321	43.146
Noise (µV^2^/Hz)	20.619	20.643	20.7773	20.797	20.525
Power (µW)	2.86	2.682	2.23	0.81562	4.7027
Bandwidth (kHz)	5.3297	5.2387	6.036	3.849	4.463
Area (µm^2^)	781.49	746.15	425.73	369.98	513.38
	**Set 6**	**Set 7**	**Set 8**	**Set 9**	**Set 10**
Gain (dB)	47.884	47.8685	47.867	46.66	47.07
Phase (degrees)	43.938	42.285	41.763	44.643	37.552
Noise (µV^2^/Hz)	20.567	20.557	20.55	20.472	20.413
Power (µW)	5.20835	6.73429	6.763	3.7032	22.984
Bandwidth (kHz)	4.0079	3.849	4.00793	4.667	4.2886
Area (µm^2^)	494.26	510.89	357.55	287.24	640.042

**Table 8:** Metaheuristic algorithm results vs. ANN-assisted goal attainment method.

	Metaheuristic Algorithm	ANN-Assisted Goal Attainment Method
Gain (dB)	41.255	47.7046
Phase (degrees)	61.96	46.321
Noise (µV^2^/Hz)	20.558	20.797
Power (µW)	2.884	0.81562
Bandwidth (kHz)	5.308	3.849
Area (µm^2^)	773.6955	369.98
